# Supplementary material for: Nonalcoholic fatty liver disease and cognitive impairment: A prospective cohort study
Source: PLoS One. 2023 Apr 14;18(4):e0282633. doi: 10.1371/journal.pone.0282633 (PMC10104321; doi:10.1371/journal.pone.0282633)
Supplement: Attachment — Submitted filename: Response to Reviewers 3-17-22.docx [file pone.0282633.s001.docx]

Response to Reviews, PONE-D-20-07431; Cushman et al., Nonalcoholic Fatty Liver Disease and Cognitive Impairment: a Prospective Cohort Study

This manuscript re-submission was severely delayed due to the pandemic, and as per the Editor instructions, I am submitting it as a new manuscript including detailed responses to the original editor and reviewer comments below. The manuscript was also updated with respect to literature review since the original submission in 2020. The authors thank the reviewers and editor for their helpful comments, and for their current consideration. Text insertions in the manuscript corresponding to specific reviewer comments are provided in blue. It is important to note that just 2 months ago, we were pleased to see a new study published from China that corroborates our finding of an age interaction in the association of NAFLD with cognitive impairment (Liiu Q., et al). We have added discussion of this.

Response to Editor Comments

1. Please describe in your methods section how capacity to provide consent was determined for the participants in this study. Please also state whether your ethics committee or IRB approved this consent procedure. If you did not assess capacity to consent please briefly outline why this was not necessary in this case.

*Response. Interviewers were trained on how to identify participants who answered questions in a way that indicated lack of comprehension. Potential participants who were able to respond to telephone questions provided verbal consent, which was followed up with written consent at an in home visit. These procedures were approved by the IRB and we added a statement on this at top of page 6.*

2. Thank you for including your ethics statement: "Institutional Review Boards at each study institution reviewed and approved the study protocol. All participants gave written informed consent to study participation."

Please amend your current ethics statement to include the full name of the ethics committee/institutional review board(s) that approved your specific study.

*Response. We added this at top of page 6.*

Once you have amended this/these statement(s) in the Methods section of the manuscript, please add the same text to the “Ethics Statement” field of the submission form (via “Edit Submission”).

*Response. done*

Response to Reviewers' comments:

Reviewer #1: This study aimed at assessing the risk of cognitive function impairment in patients with non-alcoholic fatty liver disease (NAFLD).

This topic is interesting as NAFLD cases are expected to increase in Western Countries.

Nevertheless, I would suggest some points of revision:

a. STUDY DESIGN: please summarize how follow up was performed (times of contacts), median of follow-up times.

*Response: Follow up was performed with phone call contact every 6 months after enrollment, as stated in line 104, page 5). The mean follow up time at which we sampled this nested case-control study was 3.4 years, as was stated on line 130, page 7 and in the abstract.*

b. STUDY DESIGN: It would interesting to assess the relationship between gravity scores of NAFLDS (e.g. NAFLD fibrosis score) and cognitive impairment incidence.

*Response: We have a separate detailed manuscript in progress evaluating liver fibrosis and health outcomes in REGARDS. If the reviewer and editors would allow it, we prefer not to add that to this manuscript, which already has quite a few risk factors being assessed (FLI and liver biomarkers).*

c. STATISTICAL ANALYSIS: please better underline that the analysis was conducted on subjects’ baseline characteristics. No mention is made on how categorical variables were analyzed.

*Response: We clarified on page 8 line 162 that all covariates were collected at baseline, and clarified in the statistical analysis section on page 9 line 182 that the covariates were from baseline and that multilevel categorical variables were assessed as indicator variables.*

d. RESULTS: please also report number of deaths at follow-up, including comparison in the group NAFLDs compared with non-NAFLDs.

*Total deaths in the control group excluding heavy alcohol users and not missing for NAFLD are here:*

*NAFLD positive (FLI>60): 6.9%*

*NAFLD indeterminate (20<=FLI<=60): 8.3%*

*NAFLD negative (FLI<20): 4.0%*

*These rates are not different across groups (p = 0.34). We are not sure why the reviewer asked about this in the context of our aims, so did not add it to the manuscript.*

e. TABLE 1. Please include statistical significances. Avoid redundancies between table and text.

*Response: The clinical profile of NAFLD is well characterized and table 1 is not being presented for hypothesis testing, but just to describe the cohort by NAFLD status, so p values are not appropriate to our purpose, and we did not add them. We very briefly summarized table 1 in the text and did not repeat any of the numerical data. Here is the small amount of text we believe the author is asking us to delete, but we request consideration that this be kept for the benefit of ease of reading, “Table 1 shows that those with NAFLD were more likely men, were less well educated, had adverse cardiovascular risk profiles and higher ALT and GGT. They did not differ by age, race, alcohol use, income or prevalent cardiovascular disease.”*

f. DISCUSSION: it was not evaluated in a sub analysis the cognitive domain involved. This is of relevance to distinguish symptoms of minimal encephalopathy from secondary cognitive impairment.

*Response: We believe the reviewer is asking about analyzing different cognitive tests separately. We defined our outcome of cognitive impairment using 3 tests that administered over time in order to identify an MCI-like condition which has clinical importance. Two out of 3 tests needed to be below a normed threshold to meet the case definition. Of the cases, 412 had impairment on Word List Learning (WLL) and Word List Recall (WLR), 13 on WLR and Animal Fluency, 27 on WLL and Animal Fluency, and 43 on all 3 tests. Given that the case group was selected based on joint scores on all 3 tests, it would not be valid to split the cases apart into separate groups, and any analysis of this would be underpowered. The markers we used (semantic fluency and word list learning) require a number of cognitive processes (and by implication brain networks) for successful completion including attention, memory, learning, executive function, semantic knowledge, language comprehension, language expression, and speed of processing. It would be an artificial effort to equate performance on any of the 3 markers (WLL, WLD, or Animal Fluency) to any specific cognitive ability. Since these tests are sensitive (but not necessarily specific) markers of cognitive function, they are well-suited to capturing dysfunction associated with any kind of brain dysfunction whether it be minimal encephalopathy or secondary cognitive impairment due to other neurologic factors. Finally, because we don’t have dementia classification, we are unable to define secondary cognitive impairment. We hope this information is helpful to the reviewer.*

g. DISCUSSION: The significant incidence of cognitive impairment in younger compared with older subjects it can be explained by the higher prevalence of dementia in older where the effect of NAFLDs can be hidden by other neurodegenerative factors. Please better describe the physiopathology subtending the possible toxic-neurodegenerative damage in these patients.

*Response: The reviewer refers to prevalence of dementia, however, we excluded participants with baseline cognitive impairment from this analysis so there should not be any participant with dementia at baseline to confound associations. Although droput from the study was uncommon, it is possible that older participants more often dropped out due to cognitive problems, so we missed a relationship in older people. It could also be that there were unmeasured confounders as you suggest. We cannot make a conclusion about the pathophysiology of this finding since we do not have the data to do so, but suggest replication of these findings and further study. Since the original submission, another study has observed similar findings on age differences in this association (Liu et al, reference 17), which bolsters interpretation of our findings. We added the following to the discussion of limitations on page 19, line 355, “It is possible that associations of NAFLD with cognitive impairment were not apparent in older participants due to differential loss to follow up based on cognitive outcome, or unmeasured confounders impacting on the relationship between NAFLD and cognition in older people.”*

Reviewer #2:

I was pleased to review the manuscript entitled “Nonalcoholic Fatty Liver Disease and Cognitive Impairment: a Prospective Cohort Study” by Cushman et al. Authors aimed at identifying the association between NASH (estimated through the calculated FLI) and the incidence of cognitive impairment evaluated by means of an verbal fluency test, a word list learning test and the word list recall performed by phonecall follow-up visits within the REGARDS cohort. Findings were compatible with a role of NASH in contributing to the genesis of cognitive impairment in verbal fluency, attention, immediate and delayed memory in subjects in the 45-65 age span.

The strength of the study is in the large population, but both NASH and cognitive assessment are flawed due to limitations intrinsic to the REGARDS cohort. However, statistics and results supports the hypothesis and further studies are warranted to check for the associations between NASH and cognition. Multiple corrections including for metabolic factors (acknowledged risk factors for dementia) have been performed. Results are fairly discussed and placed in the context of the current status of the literature.

*Response: We thank the reviewer for pointing out the strengths of the manuscript.*

Comments:

-We acknowledge that the topic of the link between the patient metabolic status and cognitive functioning is a serious point to pursue in epidemiological research. The main conflicting point here is that diabetes and metabolic syndrome are well known risk factors for dementia, hence authors should better specify the role of the liver in the gut-liver-brain axis that should not be interpreted just as the incapacity of a cirrhotic liver to filter gut toxins as in hepatic encephalopathy. The suffering liver could alter the production of various circulating substances that has been shown to protect brain functioning (see doi: 10.1007/s11011-015-9721-x). This would be of help in introducing the link between liver and cognition.

*Response: These are valid points. We modified the introduction to clarify the possible pathophysiology and cited the Marano paper. We also added this sentence to the final paragraph of the work, page 19, line 365, “More study is also needed to determine the mechanisms behind our observations.”*

-It would be useful to add baseline and follow up scores at neurolopsychological tests in a further table (or maybe in supplementaries) to see how enrolled subjects perform with age.

*Response: All participants had normal cognition based on a 6-item screener at baseline. The tests used to classify cognitive impairment were not necessarily administered at baseline, as described in the detailed methods article we cited that describes the nested case-control study (reference 24, Gillett et al). This manuscript focuses on a case group with a phenotype of significant cognitive impairment. The control group was free of this level of cognitive impairment. We are not sure what purpose would be served in relation to our hypothesis about liver markers and cognitive impairment by providing this level of descriptive data here so have not added it, especially given the large amount of data already provided. There are many REGARDS papers that detail the change in cognition with a variety of risk factors that the interested reader can refer to.*

-It is not clear the mean follow up time of the incident cognitive development and if the two impaired follow up tests are contigous in time.

*Response: Detailed methods for case identification have been published and cited as mentioned above (reference 24, Gillett et al). The mean time to cognitive impairment was 3.4 years as we had stated in the abstract and on page 7 line 130 of the current manuscript; perhaps the reviewer missed this. Cognitive tests were administered in staggered fashion so as to not over-burden participants during phone calls. The WLL and WLR were administered on a call staggered with the Animal Fluency test. The time to an impaired state was based on results of the most recently administered triad of tests at the time we drew the sample for this nested case-control study. This methods detail is on page 6 starting on line 117, which we edited for further clarity.*

-In the results section it would be of help to include the p values in both table 1 and 2, and model p values in table 3 and 4.

*Response: Tables 1 and 2 are descriptive tables and we were not testing hypotheses (we know the correlates of NAFLD and cognitive impairment already), so p values were not presented. In tables 3 and 4, p values for each OR would not add interpretive information to the provided confidence intervals so we did not add them. Many journals discourage indiscriminate use of such p values.*

-The language and manuscript organization should be revised in several paragraph mainly part of the abstract (37-39), the introduction (60-64, 74-75, 80-82) and the discussion section (253-254).

*We addressed the lack of clarity or grammar in the listed areas.*

-Consider the low sensitivity/specificity of the 3 neurological scales in the limitation of the study especially for younger subjects

*Response: From the neuropsychological point of view, the sensitivity and specificity of the tests we used for identifying cognitive impairment due to NAFLD is likely higher in young people because every study ever conducted has demonstrated that cognitive impairment is more prevalent in older people, mainly because they have many more pathways to cognitive dysfunction (i.e., beyond NAFLD). Nevertheless, we added information on uncertain sensitivity to detect dementia, if that is what the reviewer is referring to. We hope to have detailed dementia classification of the cohort soon. See page 19, line 353 for this added text.*
