# Supplementary material for: Nonalcoholic fatty liver disease and cognitive impairment: A prospective cohort study
Source: PLoS One. 2023 Apr 14;18(4):e0282633. doi: 10.1371/journal.pone.0282633 (PMC10104321; doi:10.1371/journal.pone.0282633)
Supplement: Attachment — Submitted filename: Response to Reviewers 10-25-22.docx [file pone.0282633.s002.docx]

**Response to Reviewers, PONE-D-22-08991**

Reviewer #1: Overall
This manuscript overall addresses a novel and interesting research question. Overall, the analysis has been well thought out and executed. I think with some changes to clarify details throughout (detailed below) and some clarity around the primary and secondary outcomes, and toning back the conclusions, this would be a publishable piece of work.

We are grateful for the effort and detailed comments from the reviewer – thank you.

Abstract
Background/aims- Would clearly indicate primary aim and secondary aims (as all outcomes are listed together currently.

We modified the abstract for clarity:

Abstract: “We studied associations of NAFLD with risk of cognitive impairment. Secondarily we evaluated liver biomarkers (alanine aminotransferase (ALT), aspartate aminotransferase (AST), their ratio, and gamma-glutamyl transpeptidase).”

Methods- Were the controls derived from the same cohort? This isn’t clear

We specified that controls were from the cohort: “587 controls were selected from an age, race and sex-stratified cohort sample of the cohort.”

Results- NAFLD ‘at baseline’ was associated with a 2.01 fold…. I think you should be transparent as you don’t have follow up information regarding est NAFLD status

We did not study risk of NAFLD over time, nor did we suggest that the best we can tell; we defined it in the methods section as being measured at baseline. The purpose of this study was to prospectively evaluate baseline NAFLD with future cognitive impairment, not change in NAFLD. The prospective design is a major strength of the study and reduces the possibility of reverse causation. Lack of data on incidence of NAFLD is a limitation of the study that is common to most observational cohort studies, and is beyond the scope of this analysis as we don’t have information to study this. If we could measure NAFLD during follow up and evaluate it as a time-varying risk factor, we would likely observe a larger association of NAFLD with cognitive impairment due to improved classification of NAFLD status. We clarified that NAFLD was measured at baseline.

Conclusion- given you only have an est of NAFLD (no liver outcomes just estimates) and this is not a generalisable cohort I think your conclusion is an overstatement of your findings. Can you re word it to reflect these points?

-We reworded the conclusions to be more tempered as suggested, “A laboratory-based estimate of NAFLD was associated with ~~contributes to~~ development of cognitive impairment,…..”

-For the information of the reviewer, the study population is quite generalizable to age-similar Black and White adults in the US. In fact, they are more similar to US Census-captured people than Black and White participants of similar age in NHANES, which is sampled purposefully to represent the general US population. Nevertheless, we added a generalizability limitation to the Discussion section as requested below by the reviewer (since we didn’t study all possible people).

Intro
Line 68/69- toxin production in chronic liver disease? Is this a likely stipulation?

We do not understand the question. In the parenthetical phrase, we present two thoughts about why liver disease might relate to cognitive function. We introduced the word, neuronal to hopefully make it more clear: “toxin accumulation might cause neuronal damage, as might lowered production of protective substances.” Line 69

Line 80- ‘the very old’ do you mean older adults? And this sentence needs to be reworded or better explained as I am not sure what you mean

This was rephrased: “In much older adults this relationship is reversed..”

Line 90- I don’t think FLI is a valid marker or NAFLD, rather a surrogate marker that has been shown to have reasonable validity in epidemiological studies. I would suggest you say it was used to ‘estimate’ NAFLD.

Agreed; the wording was changed: “The fatty liver index (FLI), a surrogate marker of NAFLD with reasonable validity [18-20], was used to estimate NAFLD.”

Line 87-90- your primary aim/ research question is not clear, can you phrase this as a primary outcome and X Y X (secondary outcomes) will also be assessed?

Thank you - We modified the text for clarity: “To test this, we investigated the relationship between NAFLD and incident cognitive impairment in a national US cohort, the REasons for Geographic And Racial Differences in Stroke (REGARDS). The fatty liver index (FLI), a surrogate marker of NAFLD with reasonable validity [18-20], was used to estimate NAFLD. Secondarily, we studied associations of individual liver disease biomarkers with cognitive impairment.”

Methods
Line 155 – can you explain why you used a cut off below 20 not 30 for ruling out FLI?

We used a value of 20 as it is more restrictive to rule out NAFLD and we wanted to minimize misclassification as much as possible to define a reference group without NAFLD. According to a validation study by Bedogni and colleagues, if FLI is less than 20 the likelihood of not having NAFLD is greater than 91%. This value was used in at least one other epidemiology study that we cited (Gastaldelli, reference 31). We added reference 19 cited just above in this sentence. We added the rationale to the sentence: “An FLI >60 suggests NAFLD (probability to have NAFLD is 78%), and FLI <20 has a high sensitivity to rule out NAFLD (probability to not have NAFLD 91%.” Line 157.

Line 165-166- can you describe how alcohol intake was measured?

This was measured by self-report as stated (now line 164).

159- there are no descriptions of the methods or tools used to measure/ height, weight, physical activity etc

We had cited the source paper for this information (to minimize text length) but are now providing more information:

-Clarified which variables were self reported: “All covariates were based on data collected at baseline. Race, alcohol drinks per week, income, physical activity level and prebaseline stroke were established by participant self-report” line 164

-Height and weight were measured with standardized methods: “Body-mass index (BMI) was calculated using baseline measured height and weight, with weight measured using a standard 136 kilogram calibrated scale and height measured with 2.5 meter metal tape measure and square, both measured without shoes.” Line 173

-Physical activity was categorized as any weekly exercise or none by response to the question, “How often each week to you exercise enough to work up a sweat.” Line 180. We note that it has been remarkable in REGARDS papers that responses to this question identify people at risk of a variety of outcomes.

As per the STROBE guidelines, it would be helpful to include a flow chart describing the participant inclusion

We agree this is important. For selection of this nested case control study sample, this diagram was already published so we now refer the reader to the source, line 139: “The flow chart describing this nested case-control sample selection was previously published. [25] In addition, we added new Figure 1 to illustrate the inclusion of participants into the analysis of the association of FLI with risk of incident cognitive impairment.

New figure legend: Fig 1: Inclusion of cases and controls in the analysis of the association of FLI with risk of incident cognitive impairment.

New text, line 207: Fig 1 shows the inclusion of cases and controls in the analysis of FLI and risk of cognitive impairment, and the proportion of each group classified with NAFLD during follow up. Few participants were heavy alcohol users; missing status for FLI related primary to missing stored blood samples.

We removed previous text in this location that referred to the numbers in this figure.

Results
Definitions for exercise, dyslipidaemia, diabetes etc have not been made clear for table 1

-We had cited a source paper for these in the methods section, but now add the above definitions of covariates, and these, to the methods section starting on line 168: “Diabetes was defined as fasting glucose ≥ 126 mg dL−1, nonfasting glucose ≥ 200 mg dL−1, or self-reported use of medications for diabetes. Dyslipidemia was defined based on definitions at REGARDS baseline as total cholesterol ≥240 mg/dL low-density lipoprotein cholesterol ≥160 mg/dL, high-density lipoprotein cholesterol ≤40 mg/dL, or self-reported current use of lipid lowering therapy.”

-A footnote was added to table 1, “* Definitions of variables can be found in the methods section.

I would consider combining Tables 1 and 2 as the variables are the same

We prefer to keep the tables separate as Table 1 only refers to the control group baseline characteristics by NAFLD status and Table 2 refers to characteristics based on presence or absence outcomes. A merged table would be a bit unwieldy and large. We only have 4 tables. If the editor prefers, we can move Table 2 to supplemental material.

Line 222- whats the definition of older age?

This referred to people older than 65 and we thought it might be obvious from the context of the sentence. We now clarify: “…there was a nearly 4-fold increased risk in those under age 65 and no association above age 65 (p for age interaction 0.03).” Line 239.

Discussion
Line 268- general ‘US’ population? The current wording is misleading

Good point. While REGARDS is a good representation of age-similar Black and White Americans, we haven’t published this evidence; we removed the word general.

Line 279- and what did this study show?

Thank you for asking. The study actually did not assess cognitive change. The sentence was edited: “only one prospective study was available, and this was a small clinic population of 331 people with NAFLD and alcoholic liver disease who were followed over 3 years for functional difficulty and did not have follow up cognitive testing or a control group, so did not address the question of the relationship of NAFLD to incident cognitive impairment.” Line 297.

Line 295- Given you adjusted for T2D, BMI and other risk factors and that did not explain your results, what does that suggest? This point is not complete

We clarified that the purpose of adjustment was to control for confounding. We also added another sentence following, “While residual confounding could be present, findings support the need to study reasons for this independent association.” Line 315

Line 310-311- this is a hypothesis? It is written as a fact and there is no reference?
Reference 47 was added.

319-320- your concluding sentence does not support the evidence you have presented in this paragraph

The sentence was removed.

325- what are ‘patients who were normal’ what is normal?

This was rephrased, “including patients with or without cognitive disorders,….” Line 345

Line 360- NAFLD – typo, and ‘strong associations’ is an overstatement as you need to state this is in a US population. I suggest you add the generalisability of your sample as a limitation further up and modify your conclusion to reflect this

-The sentence was rephrased” “In summary, this research demonstrated a tripling of the risk of incident cognitive impairment in middle-to-early-older age Black and White US residents with NAFLD.” Line 383.

-We added the requested limitation on line 372: ” Generalizability of the study sample was limited to Black and White persons in the US, so results require replication in other contexts.”

-We deleted a related sentence that was already present and was aimed at addressing this point, but didn’t use the term generalizability, “Finally, we cannot make conclusions about populations that were not studied, such as other race/ethnic groups or younger people.”

-The reviewer may also have missed that the conclusions already called for more studies including replication as a means to address generalizability and confidence in the findings.

Line 365- I think you can say liver specific outcomes rather than better classification which is unspecific

The reviewer is asking us to consider liver disease as an outcome. Our point is to consider liver disease as an exposure which is measured using better methods than we were able to apply. We tried to clarify the meaning of the sentence, “If these findings are further confirmed in studies with better classification of NAFLD than FLI….” Line 388

Line 365- NAFLD typo

Corrected

Reviewer #2: Dear Authors, I was pleased to read your manuscript. The authors wanted to see if there was a link between NAFLD (as measured by the computed FLI) and cognitive impairment as measured by a verbal fluency test, a word list learning test, and word list memory during phonecall follow-up visits in the REGARDS cohort. The findings were consistent with NAFLD playing a role in the development of cognitive impairment in participants aged 45 to 65.
Thank you for these comments.

The study's value comes in its huge sample size, although the NAFLD and cognitive assessments are inaccurate because to REGARDS cohort restrictions. The hypothesis appears to be supported by statistics and findings. In fact, the FLI is a method to calculate the risk of having hepatic steatosis, but it does not discriminate whether the cause is due to alcohol consumption or not. Even if you have corrected for this covariate, you should state this limitation of the FLI in your discussions.

We recognize that the FLI does not distinguish NAFLD from alcoholic liver disease. To address this, we had excluded participants with heavy alcohol use from all analyses of NAFLD as an exposure (defined as >14 drinks/week for men or >7 drinks/week for women) to minimize any impact of alcohol-related liver disease. We added a sentence to the limitations section on line 366: “While we excluded people with heavy alcohol use from analyses of NAFLD as an exposure, it remains possible some participants classified with NAFLD had alcohol-related liver disease.” Line 370.

Likewise, you should state that the incidence of cognitive impairment is calculated on the basis of telephone tests and is not methodologically strong.

We had cited evidence that the telephone-administered tests have validity (references 23 and 24), but nevertheless added a statement about this on line 375: “telephone-based tests may lead to misclassification.” We believe the large cohort size here and prospective study design mitigates many of the limitations, including this.

Your discussion would probably benefit from a recently published article on the subject. In the observed cohort, the clinical diagnosis of dementia was used and these results could support yours, albeit in a different population doi: 10.3389/fnagi.2021.748888.
Thank you. We added this cross-sectional study to the discussion.

Figure 1 is actually a table, which is also very impractical for the average reader, in my opinion. I suggest you revise and edit.

We think the reviewer is mistaken. Figure 1 includes 3 panels that are graphs. The reviewer must be looking at the figure legend in the text which contains tabular items describing the data (the actual figure is found elsewhere in the PlosOne system).
