# Supplementary material for: Nonalcoholic fatty liver disease and cognitive impairment: A prospective cohort study
Source: PLoS One. 2023 Apr 14;18(4):e0282633. doi: 10.1371/journal.pone.0282633 (PMC10104321; doi:10.1371/journal.pone.0282633)
Supplement: Attachment — Submitted filename: Response to reviews 1-28-23.docx [file pone.0282633.s003.docx]

**Response to Reviews Cushman et. al., PONE-D-22-08991R1**

From the editors

The authors should comment on the strengths and limitations of the study design, addressed questions related to the approach and resolve minor presentation errors

Response: Strengths were already reviewed on page 19, and limitations extensively discussed on pages 19-20 (and we have added to these based on a reviewer comment). We have responded to each reviewer concern and resolved minor presentation errors.

Journal Requirements:
Please review your reference list to ensure that it is complete and correct. If you have cited papers that have been retracted, please include the rationale for doing so in the manuscript text, or remove these references and replace them with relevant current references. Any changes to the reference list should be mentioned in the rebuttal letter that accompanies your revised manuscript. If you need to cite a retracted article, indicate the article’s retracted status in the References list and also include a citation and full reference for the retraction notice.

Response: References were checked. We did not cite retracted papers to our knowledge and did not add new references.

Reviewer Comments to the Author

Reviewer #3: The study by Cushman, et al. investigated the relationship the associations of NAFLD with risk of cognitive impairment using data from a prospective cohort study. The strengths of the study are the relatively large sample size and prospective cohort design. However, I have several concerns regarding study design and presentation of the data.

Major comments:
1. My major concern is that “NAFLD” is defined based on FLI, a non-invasive index calculated from waist circumference, body mass index, levels of triglycerides and GGT, and not based on validated imaging measures or a clinical diagnosis. Thus the reported association likely reflects the effect of metabolic syndrome (obesity and dyslipidemia) on cognitive decline, rather than the effect of NAFLD per se. The fact that the authors found no similar association of liver function tests (ALT, AST) with cognitive impairment underscores the problem. While FLI has been shown to have reasonable accuracy in discriminating between NAFLD and non-NAFLD in some settings, I think it reflects a metabolic risk profile rather than disease per se.

Response: We acknowledge the great point of the new reviewer and also agree that FLI is reasonably validated. We had included related comments about classification of NAFLD in the previous submission. We agree that metabolic syndrome and NAFLD will frequently co-occur and it may be impossible to tease apart in terms of what the causal pathway might be, even if we had a gold standard measure of NAFLD; the issue would be the same. In addition, although the FLI has components of metabolic syndrome in its formula, GGT, a biomarker for selected liver disorders, is not a component of metabolic syndrome. We adjusted for metabolic factors in the multivariable analysis, but this doesn’t mean there isn’t residual confounding. We also want to mention that individual measures of liver function do not adequately reflect NAFLD, so lack of associations for individual aminotransferase levels does not mean that the findings on FLI are invalid. For example, serum aminotransferase levels, which are often elevated in liver injury, are not specific biomarkers for NAFLD, and their levels can be normal in up to 25% of individuals with NAFLD (PubMed ID 31937252). Finally, AST/ALT >2 was indeed related to risk of cognitive impairment.

We have added mention of the possibility of residual confounding by metabolic syndrome and the close connection of NAFLD with metabolic syndrome in the limitations section of the paper, page 19, line 370 in the tracked doc, “FLI presence may also relate closely to metabolic syndrome, and as such it would be difficult to separate the impacts of NAFLD and metabolic syndrome on risk of cognitive impairment. While we adjusted for factors involved in metabolic syndrome, we cannot say definitively that NAFLD is causal for the observed associations.”

2. Definition of cognitive decline (p. 6, lines 122-123): the authors state incident cognitive impairment was defined as impaired scores on at least two of the three follow-up tests, which were performed “every two years during follow-up” (lines 118-119). While cases and controls had similar mean age at baseline (Table 2), was there any difference in the length of follow-up between the two groups or the age at diagnosis/last follow-up? This information needs to be provided in Table 2 or the Methods.

Response: Test scores comprising the case definition for cognitive impairment were normed for age, which is why cases and controls have similar age. We selected the case and control groups based on data available in 2011 for follow up to their most recent cognitive test battery prior to this date (mean follow up 3.4 years). Any difference between cases and controls for follow up time would not seem relevant in this time span as all included participants had to have follow up cognitive assessment and these were done 2 years apart. We are unclear why age at last follow up would inform interpretation of the results. Based on the study design that age would be similar in the two groups.

3. Statistical analyses: can the authors provide more information about the weights used in the analysis?

Response: We added text on page 9 of the tracked doc at the bottom (line 186) to clarify this: “Baseline participant characteristics by NAFLD status and case control status were tabulated with weighting based on age group, sex, race to account for the stratified control sample selection so that the characteristics would reflect those in the larger REGARDS cohort.”

4. Tables: please add p-values for comparison of the groups (of at least standardized mean differences). While some journals do not require p-values when presenting baseline characteristics, it is very difficult to detect important differences without scrutinizing the entire table and doing some back-of-the-envelope calculations. For example, the authors state that the groups with and without NAFLD (Table 1) did not differ by race; however, the percentages of Black subjects in the two groups were 39 and 29, which is quite a large difference.

Response: We appreciate the reviewer pointing out the difference in race between those with and without NAFLD. It was an error in the text for us to say these prevalences were similar and we have edited this. We prefer to provide descriptive text only and avoid the use of p-values in the baseline characteristics table for several reasons. First, we are not testing hypotheses here but just describing the data. Second, statistical inference is not a valid method for determining the possibility of confounding in later analyses and may provide misleading information regarding this question. Third, statistical inference measures in a baseline characteristics table address the likelihood that a study exposure and a specific covariate are related in the underlying population; this question can be tangential to the study hypotheses and potentially distracting. Fourth, the use of statistical inference measures in a baseline characteristics table involves multiple comparisons and is likely to generate false positive associations.

5. Tables – categorical characteristics should be presented as number (%), not only %.

Response: We elected to show percents since the data is weighted to the full cohort so there would then be 2 numbers and 2 percentages in each cell (one for the nested sample and one for the weighted values reflecting the full cohort). We hope the reviewer can agree this is simpler and preferable.

Minor comments:

6. Page 4, lines 67-68: “imply considered, toxin accumulation might cause neuronal damage, as might lowered production of protective substances.” Fragment/incomplete sentence. Please revise.

Response: apologies for this! We revised to, “…. pathways of an association are unclear. Simply considered, toxin accumulation …..”

7. Page 10, line 208: “group classified with NAFLD during follow up”. I am confused. I thought the authors stated that NAFLD was only assessed at baseline, not follow-up.

Response: This is correct. The phrase, “during follow up” was removed.
